# Arginine is a protective factor for risk and prognosis of gastric cancer: an observational and Mendelian randomization study

**DOI:** 10.3389/fonc.2026.1898128

**Published:** 2026-09-03

**Authors:** Yuanyuan Wang, Yutong Xing, Zhitu Zhu

**Affiliations:** 1 Jinan University, Guangzhou, Guangdong, China; 2 Department of Oncology, The First Affiliated Hospital of Jinzhou Medical University, Jinzhou, China; 3 Liaoning Provincial Key Laboratory of Clinical Oncology Metabonomics, Institute of Clinical Bioinformatics, Cancer Center of Jinzhou Medical University, The First Affiliated Hospital of Jinzhou Medical University, Jinzhou, China

**Keywords:** amino acids, arginine, gastric cancer, prognosis, risk

## Abstract

**Background:**

Previous research has indicated that certain amino acids possess anti-tumor properties. However, the impact of blood amino acid levels on the risk of gastric cancer remains uncertain, and a comprehensive analysis examining the association between blood amino acids and the prognosis of gastric cancer is lacking.

**Methods:**

We performed a Mendelian randomization analysis to explore the link between 22 blood amino acids and gastric cancer risk. We also assessed how these amino acids affect gastric cancer prognosis using Cox regression analyses and visualized this with Kaplan-Meier survival curves. A nomogram prognostic model was developed, and its predictive value was evaluated using ROC curves. Additionally, we examined the effects of a key amino acid on cell proliferation and gene expression in gastric cancer cells through CCK-8 assays and transcriptomic sequencing, followed by GO and KEGG pathway analyses to understand the molecular regulation of responsive genes.

**Results:**

Our study revealed a significant negative correlation between arginine levels and the risk of gastric cancer. Moreover, only arginine emerged as an independent prognostic factor for gastric cancer, regardless of age, sex, stage, differentiation, and other amino acids. Furthermore, the nomogram prognostic model, incorporating arginine, sex, age, differentiation, and stage, demonstrated a robust prognostic ability. The area under the curve (AUC) for predicting 1-year, and 3-year survival rates were 0.949 and 0.962, respectively. The CCK-8 analysis showed that higher arginine levels inhibited gastric cancer cell growth. Additionally, GO and KEGG enrichment profiling revealed that the identified DEGs were primarily involved in metabolism pathway and protein-binding function.

**Conclusion:**

Arginine is a protective factor for the risk and prognosis of gastric cancer.

## Introduction

Gastric cancer is a widely prevalent malignancy and is considered one of the most common causes of cancer-related mortality on a global scale (1). Consequently, it is imperative to investigate the various factors that influence the incidence and prognosis of gastric cancer. Metabolic reprogramming is a pivotal factor in maintaining aberrant proliferation within the nutrient-constrained milieu of tumor cells (2). Notably, recent investigations have increasingly focused on amino acid metabolism (3–5). Studies have revealed that specific amino acids possess the capacity to influence anti-tumor immunity (6, 7). However, the impact of blood amino acid levels on the susceptibility of gastric cancer remains unclear, and there is a lack of comprehensive research analyzing the relationship between 22 amino acids and the prognosis of gastric cancer.

The genetic epidemiological method called Mendelian randomization (MR) utilizes single nucleotide polymorphisms (SNPs) that display robust associations with the exposure as instrumental variables (IVs) to estimate the potential causal relationship between the exposure and the outcome (8–10). By assuming that genotypes are randomly assigned during gamete formation, the implementation of the IV model effectively addresses the problem of confounding in observational studies, specifically the bias caused by unmeasured confounders on causal inference (11).

This study employed the MR method to examine the association between 22 amino acids present in human blood and the risk of gastric cancer. Next, we examined the clinical relevance of these candidate amino acids using real-world patient data. Finally, transcriptomic profiling and functional assays were conducted to explore the biological mechanisms of the candidate amino acid in gastric cancer progression. Through this integrated approach, we aimed to clarify the causal and prognostic roles of specific amino acid metabolism in gastric cancer.

## Materials and methods

### Study design

**Figure 1:**
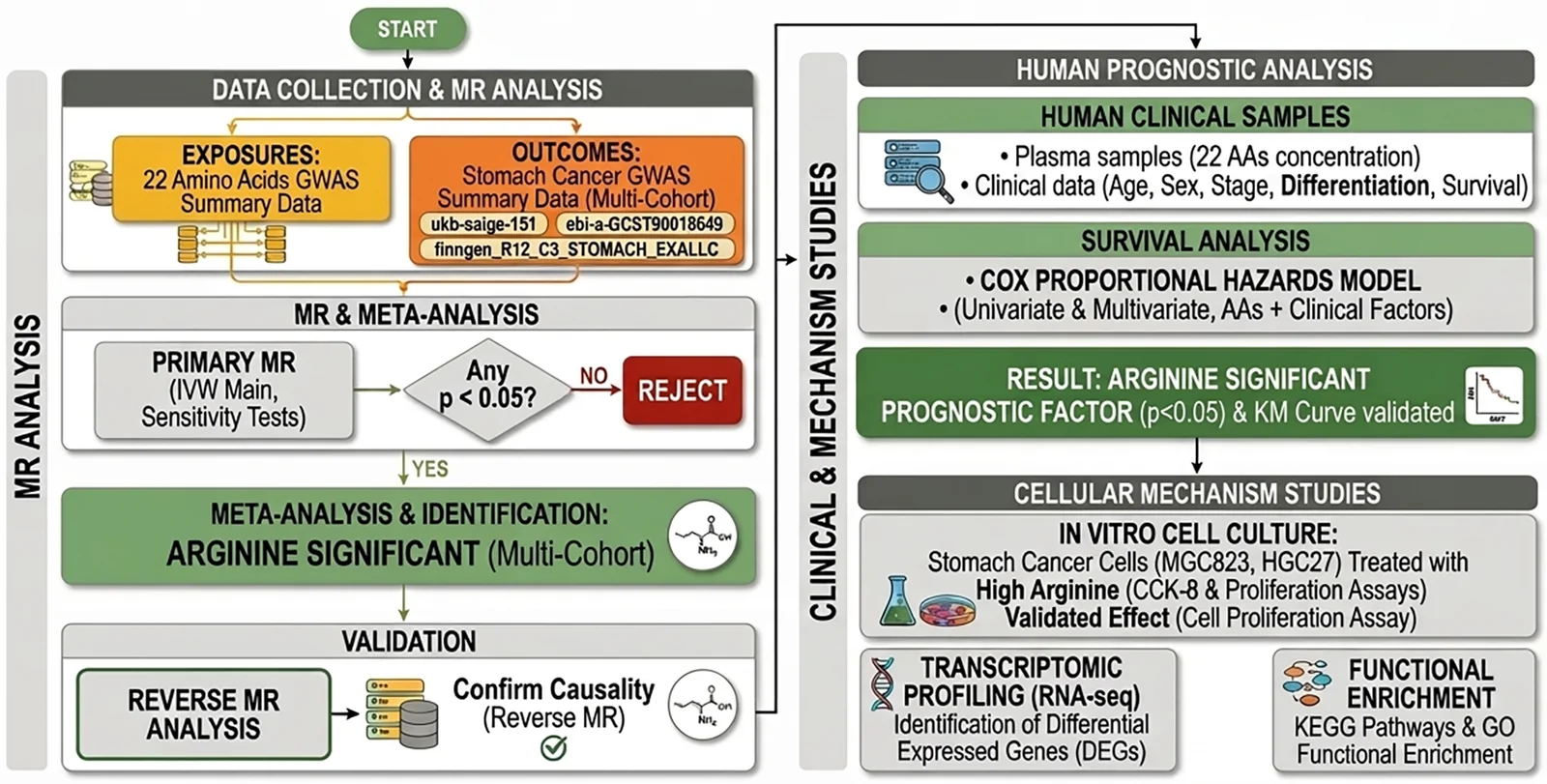
Study design.

In this study, we used the inverse-variance weighted (IVW) method to assess the causal links between 22 amino acids and gastric cancer risk in three cohorts. Statistically significant candidates from any cohort underwent a random-effects meta-analysis. Amino acids maintaining significance were deemed genuinely associated with cancer risk. To confirm these findings, we used various Mendelian randomization (MR) methods and reverse MR analysis to exclude reverse causality. We also measured plasma levels of these amino acids in gastric cancer patients. Cox regression analyses identified arginine as the only amino acid independently linked to patient prognosis, remaining significant even after adjusting for clinical factors like age, sex, tumor stage, and differentiation. To investigate cellular mechanisms, gastric cancer cells were cultured in high-arginine conditions to assess proliferation. RNA sequencing at 48 hours post-treatment identified key differentially expressed genes, further analyzed using KEGG pathway and GO functional enrichment. The specific design is shown in **Figure 1**.

### Materials and reagents

The human gastric cancer cell lines HGC-27 and BGC-823 used in this study were stored and maintained in our laboratory. Human gastric cancer cell lines, HGC-27 and BGC-823, were originally obtained from the Cell Bank of the Chinese Academy of Sciences (Shanghai, China) and Shanghai Beyotime Biotechnology, respectively. The RPMI-1640 medium and TRIzol reagent for total RNA extraction were obtained from Thermo Fisher Scientific. Fetal bovine serum, penicillin-streptomycin, and the Cell Counting Kit-8 reagent were sourced from Abbkine Scientific. Analytical grade exogenous L-arginine was procured from Beijing Solarbio Science & Technology.

### Data sources

The genome-wide association study (GWAS) datasets for blood 22 amino acids (**Supplementary Table 1**) were obtained from the IEU Open GWAS database (https://gwas.mrcieu.ac.uk/).Three gastric cancer cohorts—’ebi-a-GCST90018849’, ‘finngen_R12_C3_STOMACH_EXALLC’, and ‘ukb-saige-151’—were obtained from the IEU OpenGWAS database (https://gwas.mrcieu.ac.uk/), FinnGen database (https://www.finngen.fi/en), and UK Biobank database (https://www.ukbiobank.ac.uk/), respectively. Crucially, all datasets utilized in this study were restricted to populations of European ancestry.

In this study, we conducted a retrospective analysis of archival data from 83 gastric cancer patients treated at the First Affiliated Hospital of Jinzhou Medical University. The dataset comprised serum amino acid levels, comprehensive clinicopathological records, and historical follow-up data. Plasma amino acid analyses were performed on blood samples collected after a minimum 12-hour overnight fast. Baseline tumor markers, including carcinoembryonic antigen (CEA), carbohydrate antigen 19-9 (CA19-9), and carbohydrate antigen 72-4 (CA72-4), were also evaluated. Importantly, all blood samples were obtained prior to the commencement of any anti-tumor therapy. The specific detection method of amino acids is as described in our previous article (12). This study was approved by the institutional ethics committee of The First Affiliated Hospital of Jinzhou Medical University (No.202398).

### Selection of instrumental variables

In the current Mendelian randomization study, SNPs that displayed a statistically significant association with blood 22 amino acid at the genome-wide level of significance (P value < 5 × 10 – 6) and exhibited no linkage disequilibrium (LD) with other SNPs (r2<0.001 within a clumping window of 10000 kb) were selected as instruments for these blood amino acids. SNPs that were associated with the outcome (P<5 × 10 – 5) were also excluded. Additionally, we conducted an MR-PRESSO analysis and employed the MR-PRESSO outlier test methodology to eliminate outlier SNPs (13). Furthermore, the Steiger test approach was utilized to identify SNPs exhibiting a stronger association with the outcome variable compared to the exposure variable, and these SNPs were also excluded from the analysis. These selected SNPs were then utilized as instrumental variables for the blood amino acid analysis. However, for the reverse Mendelian randomization analysis, instrumental variables related to gastric cancer were chosen with an P value < 5 × 10 ^– 5^, while other setting conditions remained unchanged. To reduce weak instrument bias, each IV’s strength was evaluated using the F-statistic (F = β²/SE²). All chosen IVs had F-statistics over 10, showing minimal weak instrument bias risk.

### Mendelian randomization analysis

The Two-sample MR analyses were conducted using R (version 4.3.0) and the “ MendelR” package. The primary MR analysis method employed in this study was the IVW method. Additionally, the Weighted Median, Weighted Mode, and MR-Egger methods were applied. Sensitivity analyses, such as Heterogeneity (Cochran’s Q statistics), Horizontal Pleiotropy (MR-Egger intercept analysis), and Leave-One-Out analysis, were also performed.

### Cell culture

The study used two human gastric cancer cell lines, BGC-823 and HGC-27, cultured in RPMI-1640 medium with 10% fetal bovine serum and 1% penicillin-streptomycin. Cells were incubated at 37 °C with 5% CO2. When 80%–90% confluent, cells were passaged with 0.25% trypsin-EDTA. Experiments utilized cells in the logarithmic growth phase.

### Cell proliferation assay

Cell proliferation assays using a CCK-8 kit were conducted to examine arginine’s effect on gastric cancer cells. BGC-823 and HGC-27 cells were seeded in 96-well plates at 3 × 10^3 cells/well. After 24 hours of incubation at 37 °C with 5% CO2, the supernatant was removed. Cells were then treated with RPMI-1640 medium containing different arginine concentrations, with three replicates per group. At 24, 48, and 72 hours, 10 µL of CCK-8 reagent was added, followed by a 2-hour incubation. Absorbance at 450 nm was measured to determine cell viability.

### Transcriptomic sequencing

BGC-823 cells were treated with a sublethal concentration of arginine (12.5 mM) for 48 hours. Cell pellets from both treatment and control groups were collected, and total RNA was extracted using TRIzol reagent. The RNA samples were then sent to LC-Bio Technology Co., Ltd. for quality control, library construction, and high-throughput transcriptomic sequencing. Differentially expressed genes (DEGs) were identified with thresholds of |log2FC| > 1 and adjusted P < 0.05.

### Other data analysis

Summary estimates from the random-effects meta-analysis were calculated using the ‘MendelR’ package. Kaplan-Meier method was used to draw the survival curve. Univariate and multivariate COX regression analysis was utilized the “survival” package. The results were then presented in a forest plot. The ROC curve was plotted using the “timeROC” package. Using the “rms” package to plot nomogram and calibration graph, the more the calibration curve of the nomogram fits the standard curve, the higher the predictive power.

### Statistical significance

Univariate and multivariate COX analysis also showed that P < 0.05 was statistically significant. Kaplan-Meier method was used to draw the survival curve, and the Log-rank test was used for comparison, with P < 0.05 as statistically significant. Other statistical analyses also considered P < 0.05 as statistically significant. Relevant analyses were performed using R software (version 4.3.0).

## Results

### Two-sample MR analysis of amino acids and risk of gastric cancer based on IVW method

**Figure 2:**
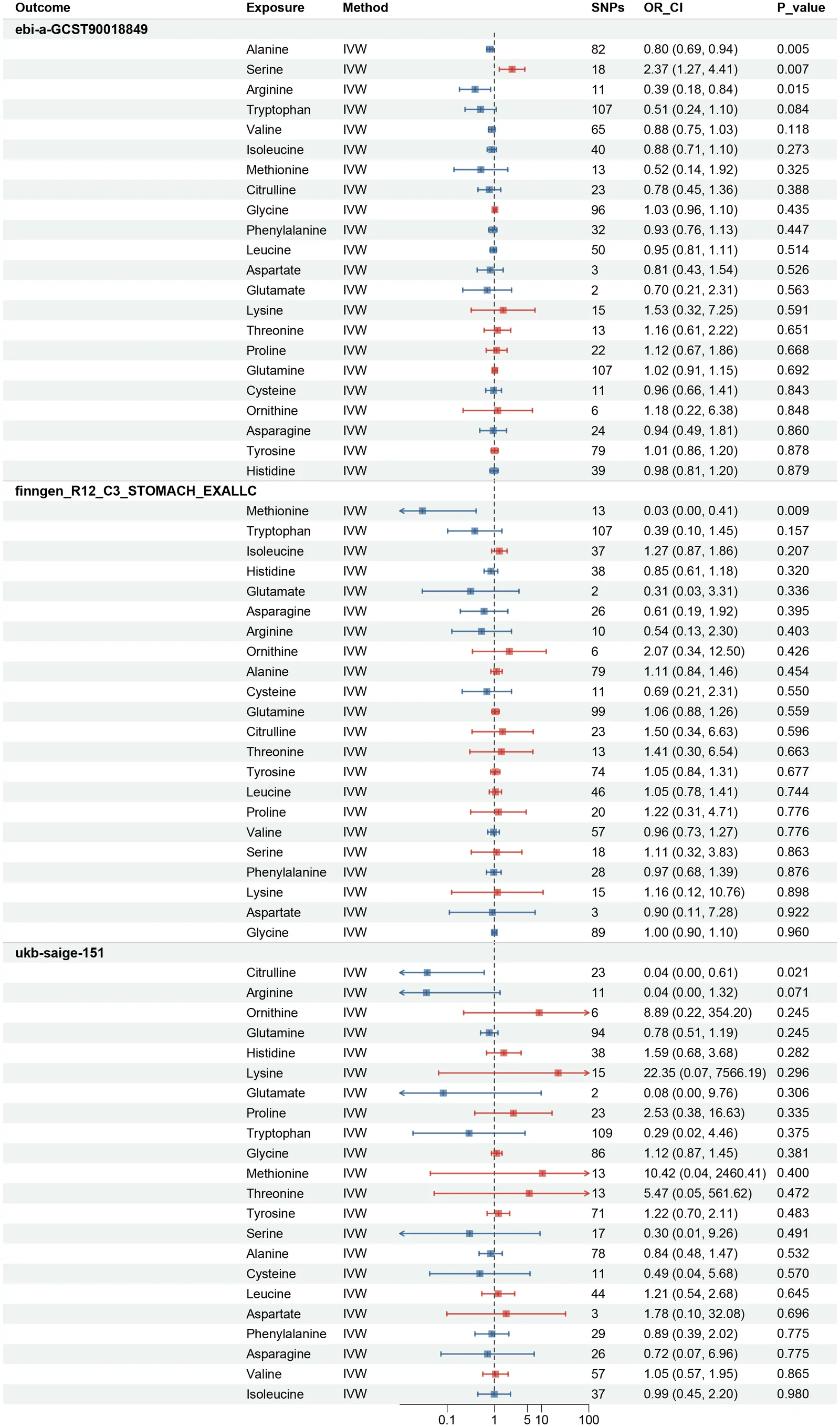
Two-sample MR analysis of amino acids and risk of gastric cancer based on IVW method.

Using the IVW method, we examined the causal links between 22 blood amino acids and gastric cancer risk. In the ebi-a-GCST90018849 cohort, arginine and alanine were negatively linked to gastric cancer risk, while serine was positively linked (P < 0.05) (**Figure 2**). In the finngen_R12_C3_STOMACH_EXALLC cohort, methionine showed an inverse correlation with gastric cancer risk (P < 0.05) (**Figure 2**). The ukb-saige-151 cohort analysis revealed that citrulline was associated with a reduced risk of gastric cancer (P < 0.05) (**Figure 2**).

### Meta-analysis results

**Figure 3:**
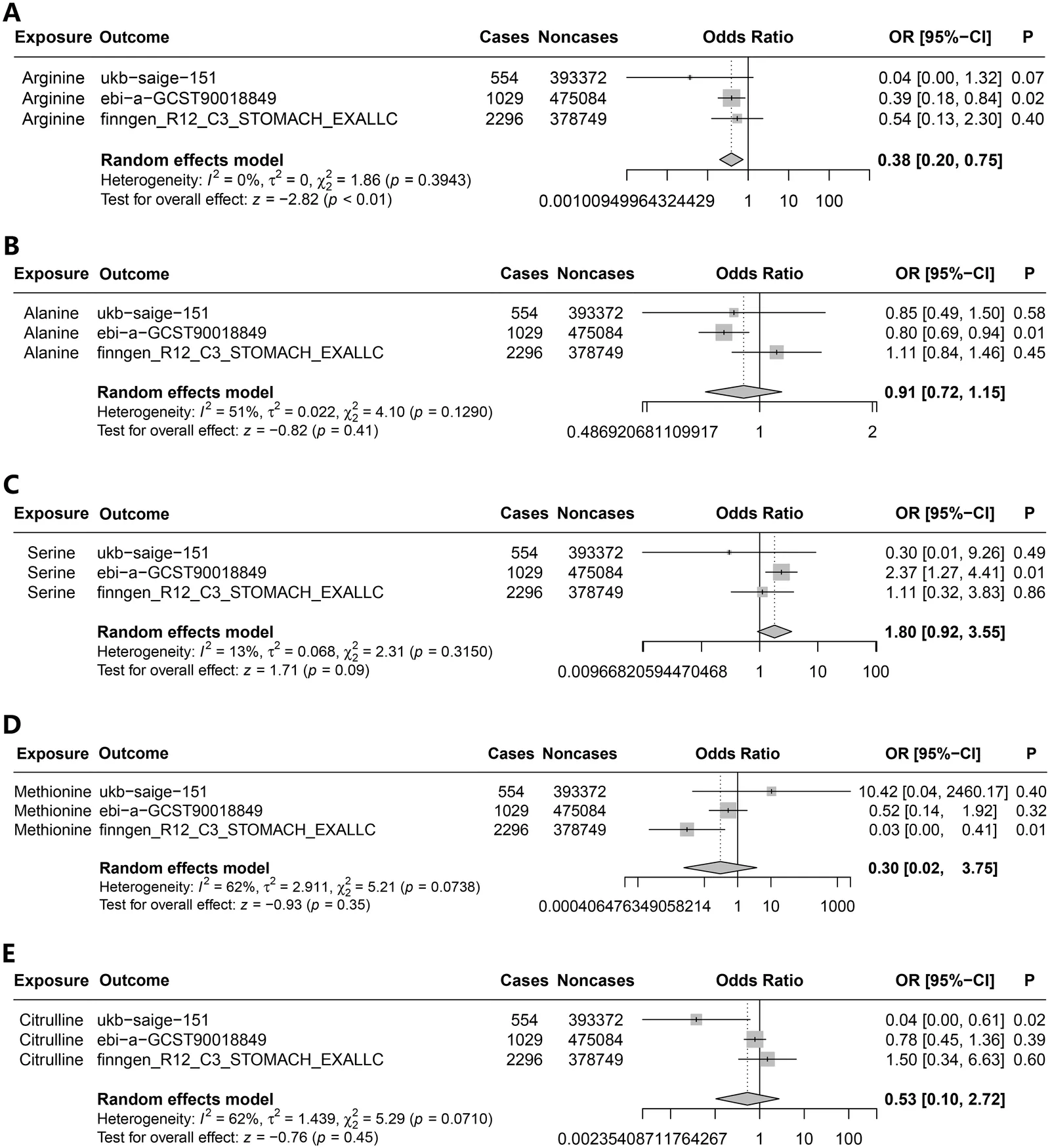
Meta-analysis of the causal associations between candidate amino acids and gastric cancer risk. **(A)** Arginine, **(B)** Alanine, **(C)** Serine, **(D)** Methionine, and **(E)** Citrulline.

For each amino acid for which the inverse-variance weighted (IVW) Mendelian randomization results demonstrated statistical significance in at least one individual cohort, a random-effects meta-analysis was conducted to aggregate the IVW estimates obtained from the three separate gastric cancer cohorts. The meta-analysis ultimately indicated that only arginine sustained a statistically significant association (**Figure 3**).

### Two-sample MR analysis of arginine and gastric cancer risk across three cohorts using four methods

**Figure 4:**
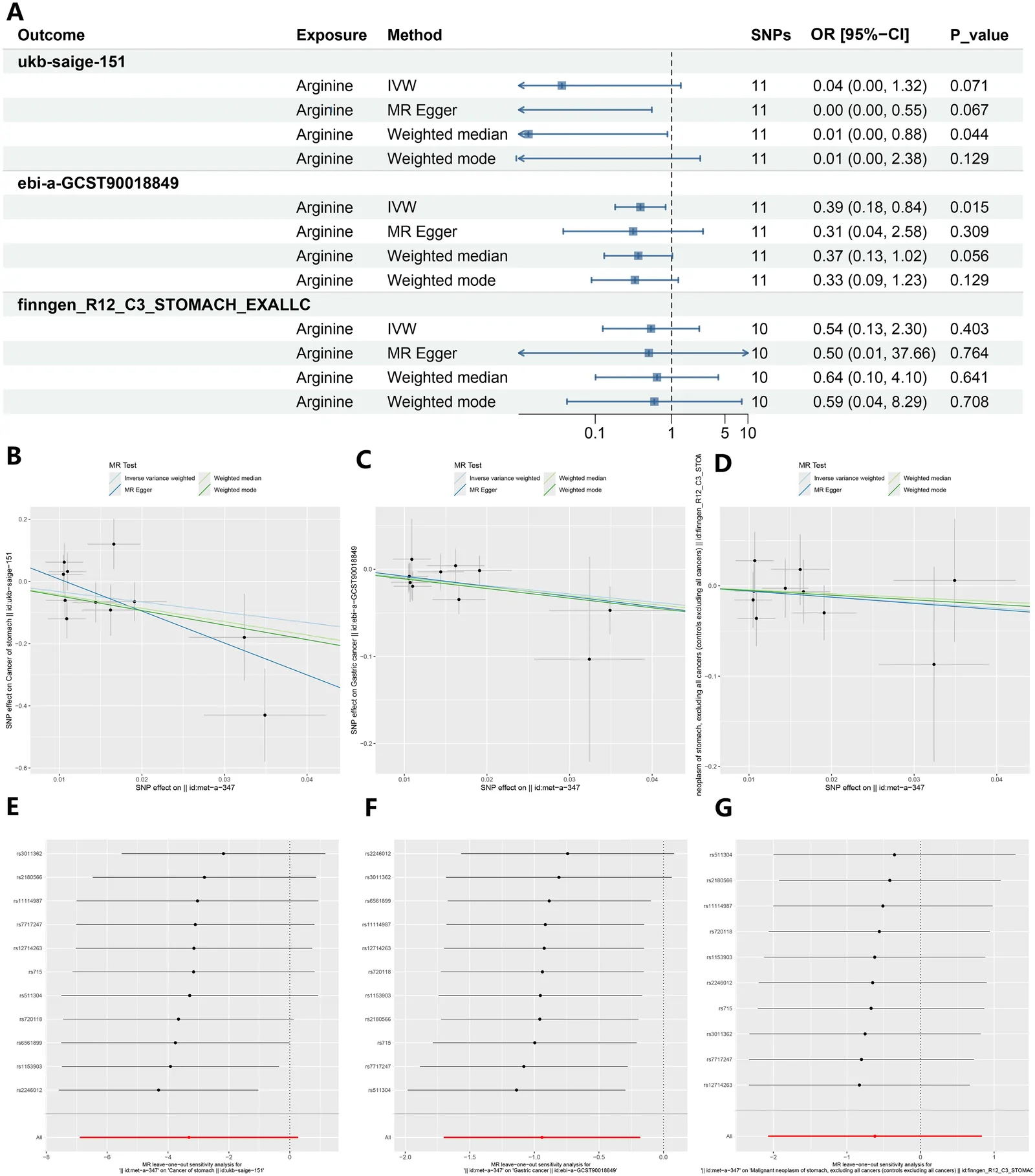
Two-sample MR analysis of arginine and gastric cancer risk across three cohorts using four methods. **(A)** Forest plots representing the MR estimates and 95% CI values of the causal effects of arginine. **(B–D)** Gene prediction of the association between arginine levels and risk of gastric cancer based on different MR methods. **(E–G)** Leave-one-out analysis results of arginine, alanine, and serine.

Our analysis of three gastric cancer cohorts consistently showed that higher arginine levels were linked to a lower risk of gastric cancer (PIVW < 0.05). All four Mendelian randomization methods produced odds ratios below 1 (**Figure 4A****),** confirming an inverse relationship between arginine and gastric cancer risk (**Figures 4B–D**).

The leave-one-out analysis indicated that a single SNP does not affect the relationship between arginine, alanine, serine, and gastric cancer risk (**Figures 4E–G**). Additionally, the sensitivity analysis results, including the MR-Egger Intercept p-value and the Cochran Q test p-value, were all greater than 0.05. Detailed results can be found in **Supplementary Material 1.**

### Reverse MR analysis of arginine and risk of gastric cancer

MR analysis of the causal relationship between gastric cancer and blood arginine showed that gastric cancer did not affect the contents of blood arginine (**Table 1**).

**Table 1:** Reverse MR analysis of arginine and risk of gastric cancer.

id.exposure	Outcome	Method	NO.SNPs	OR (95%CI	P value
ukb-saige-151	Arginine	IVW	7	1.00 (0.99-1.01	0.67
ebi-a-GCST90018849	Arginine	IVW	10	1.00 (0.99-1.02)	0.63
finngen_R12_C3_STOMACH_EXALLC	Arginine	IVW	8	1.00 (0.98-1.01	0.74

### Prognostic value of circulating plasma arginine in gastric cancer

**Figure 5:**
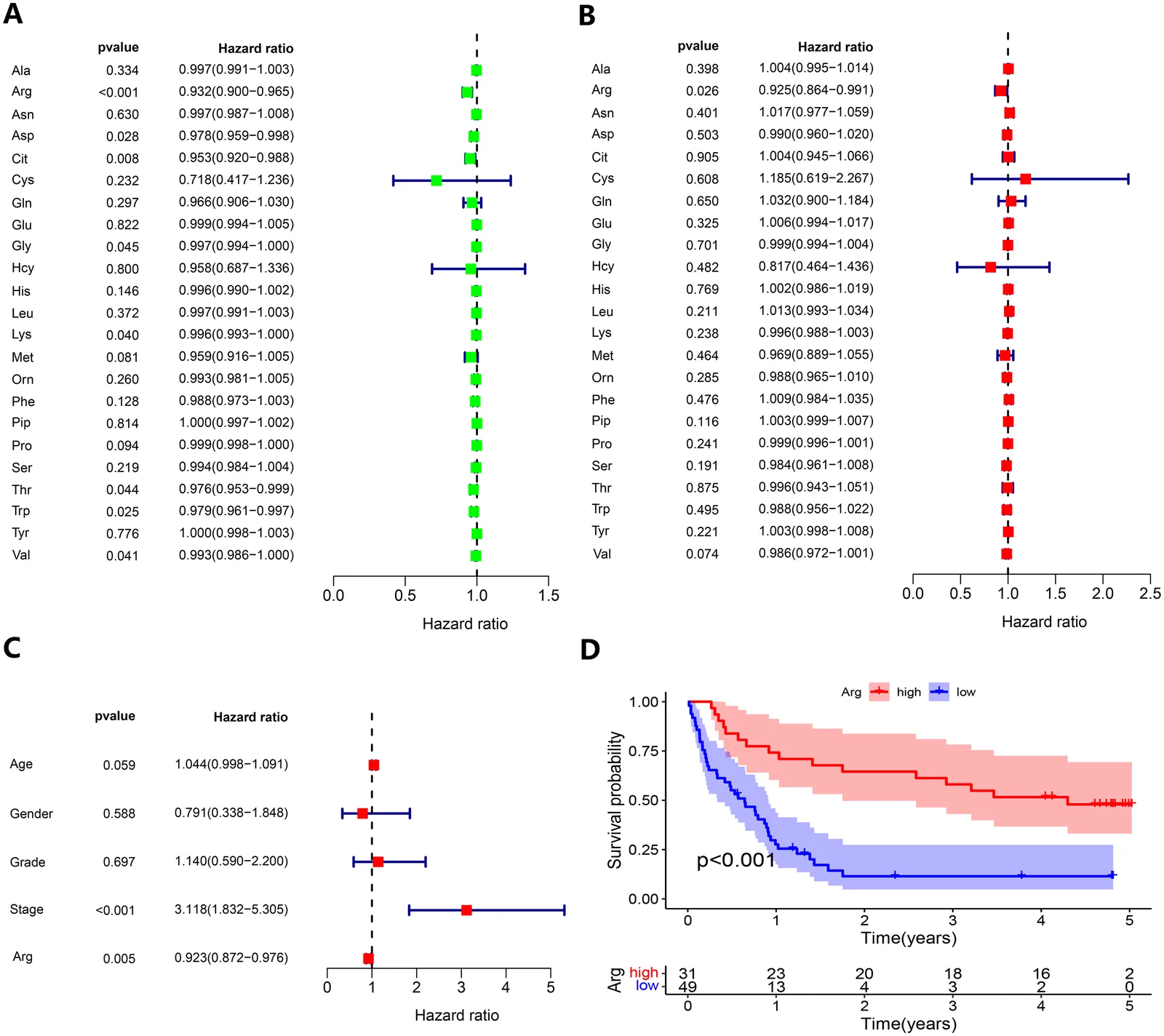
Prognostic value of circulating plasma arginine in gastric cancer. **(A)** Univariate Cox regression analysis of the prognostic significance of the 22 blood amino acids. **(B)** Multivariate Cox regression analysis evaluating the independent prognostic value of the 22 blood amino acids. **(C)** Multivariate Cox regression analysis of the association between arginine levels and patient prognosis, adjusted for age, sex, tumor differentiation, and TNM stage. **(D)** Kaplan–Meier survival curves comparing gastric cancer patients with high versus low plasma arginine levels.

Univariate Cox regression analysis identified eight amino acids, including arginine, as significantly linked to gastric cancer prognosis (P < 0.05) (**Figure 5A**). Multivariate Cox analysis further confirmed arginine as the only independent prognostic marker (**Figure 5B**), unaffected by other amino acids or clinical factors like age, sex, tumor differentiation, and TNM stage (P < 0.05) (**Figure 5C**). Kaplan-Meier survival curves showed that patients with high arginine levels had better outcomes than those with low levels (**Figure 5D**).

### Arginine based nomogram prognostic model

**Figure 6:**
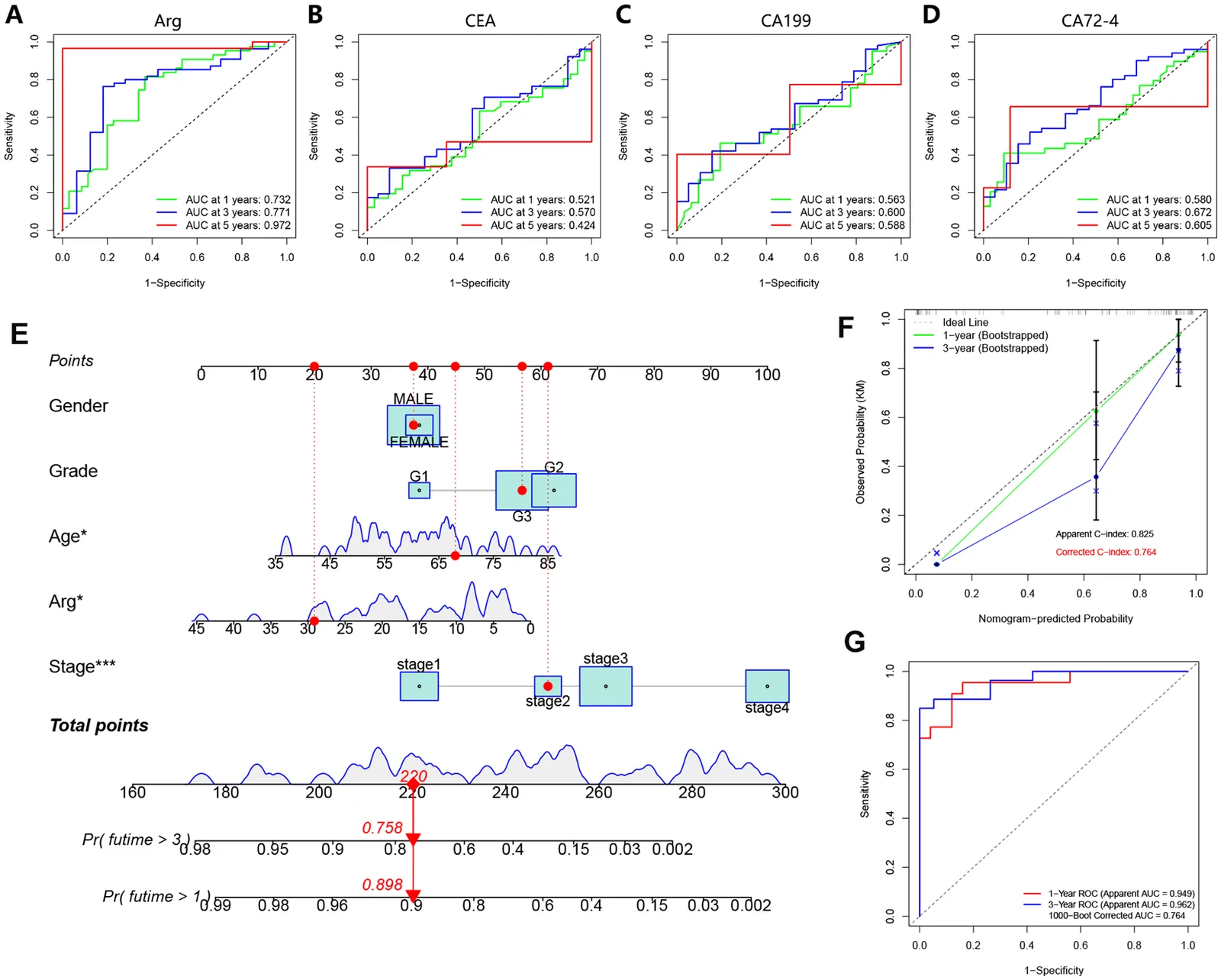
Arginine based nomogram prognostic model. **(A–D)** ROC curve of negative value of arginine, CEA, CA-199, CA-724 and 1-, 3-, 5-year survival of gastric cancer. **(E)** Nomogram prognostic model. **(F)** Calibration curves of the nomogram evaluated using 1,000-bootstrap validation. **(G)** Time-dependent ROC curves annotated with the global optimism-corrected C-index (AUC = 0.764).

The AUC curves for arginine, CEA, CA199, CA72-4, and prognosis were plotted. The AUC values for negative value of arginine at 1-year, 3-year, and 5-year intervals were 0.732, 0.771, and 0.972, respectively (**Figure 6A**). The AUC values for CEA were 0.521, 0.570, and 0.424, for CA199 were 0.563, 0.600, and 0.588, and for CA72–4 were 0.580, 0.672, and 0.605 at 1-year, 3-year, and 5-year intervals (**Figures 6B–D**). We also constructed a nomogram model based on age, sex, differentiation, stage, and arginine to better predict the prognosis of patients (**Figure 6E**). Internal validation using 1,000-bootstrap resampling showed strong calibration, with 1-year and 3-year curves closely matching the ideal line (**Figure 6F**). The apparent AUCs for 1-year and 3-year OS were 0.949 and 0.962 (**Figure 6G**), respectively, while the overall 1,000-bootstrap optimism-corrected C-index (AUC) for the global model was maintained at 0.764 (**Figures 6F, G**), indicating the nomogram’s reliable discrimination and generalizability after adjusting for overfitting.

### Arginine inhibits the proliferation of gastric cancer cells

**Figure 7:**
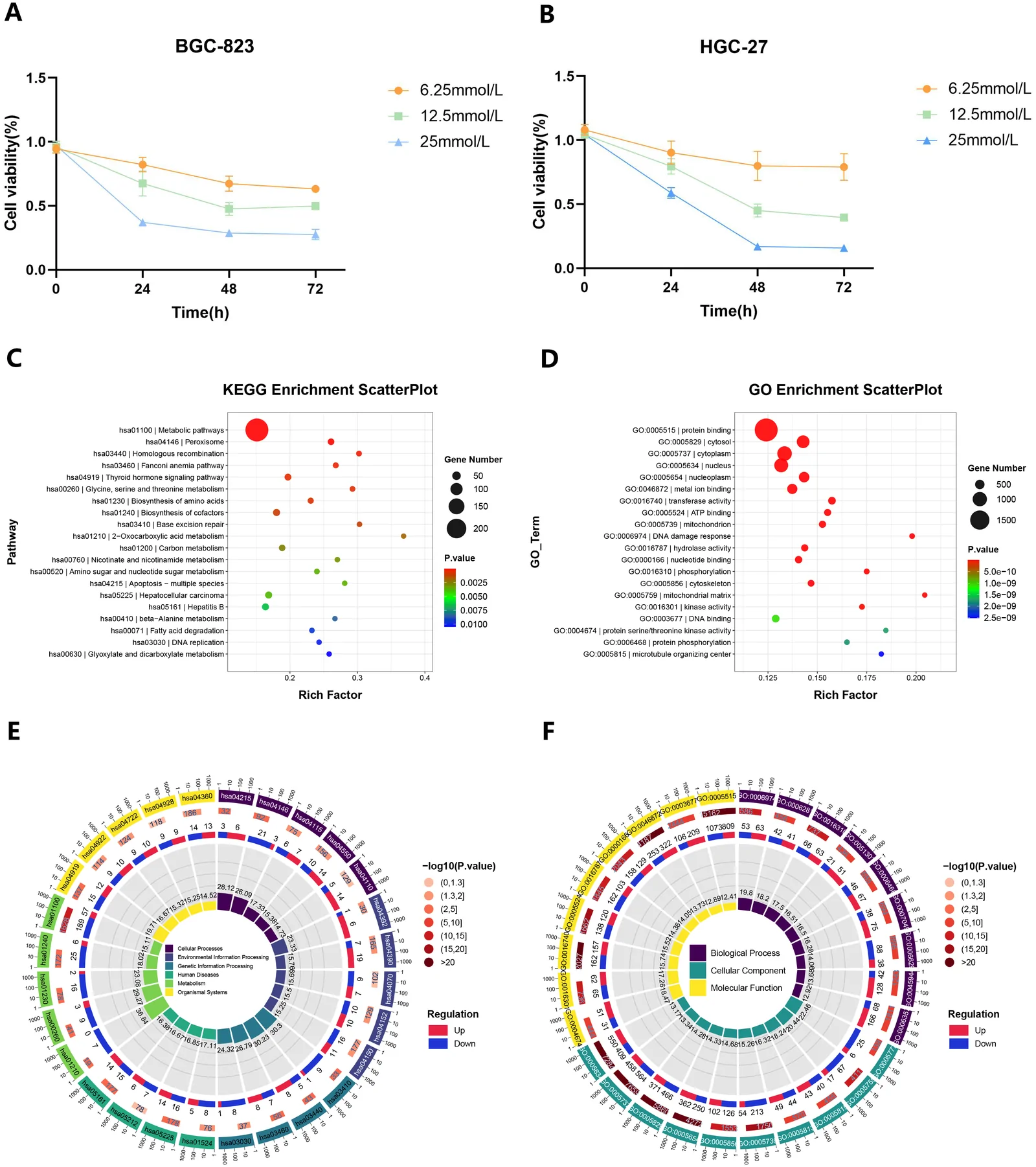
Arginine inhibits the proliferation of gastric cancer cells. **(A, B)** CCK-8 proliferation assay was used to detect the effect of different concentrations of arginine on the proliferation activity of gastric cancer cells BGC-823 and HGC-27. **(C)** KEGG pathway enrichment analysis of DEGs. **(D)** GO functional enrichment analysis of DEGs. **(E)** Enrichment circle plot of KEGG signaling pathways for DEGs. **(F)** Enrichment circle plot of GO terms for DEGs.

Previous studies indicate that arginine significantly inhibits growth and induces apoptosis in gastric cancer cells at concentrations between 2 to 32 mM (14). To explore the underlying molecular mechanisms, this study exposed gastric cancer cells to varying concentrations of exogenous arginine in RPMI-1640 medium. A preliminary CCK-8 assay confirmed a concentration-dependent growth pattern (**Figures 7A, B**), leading to the selection of 12.5 mM exogenous arginine for RNA-seq analysis. Transcriptomic profiling showed that arginine-responsive genes were mainly involved in metabolism pathways and protein binding functions (**Figures 7C, D**). The KEGG and GO circle plots illustrated the enrichment significance and functional categories of differentially expressed genes, highlighting the distribution of up-regulated (red) and down-regulated (blue) genes (**Figures 7E, F**).

## Discussion

The metabolic process of amino acids plays a crucial role in tumor cells, as they utilize amino acids for their proliferation, invasion, and immune evasion (15). However, a dearth of research exists regarding the examination of the correlation between blood amino acids and the susceptibility to gastric cancer, as well as the comprehensive analysis of the association between blood amino acids and the prognosis of gastric cancer.

In this study, the IVW method was initially employed to examine the correlation between 22 amino acids and the susceptibility to gastric cancer. A subsequent meta-analysis, which aggregated Mendelian Randomization (MR) estimates from three independent gastric cancer cohorts, revealed a significant association between arginine levels and a decreased risk of gastric cancer. Importantly, additional MR analyses employing alternative models demonstrated strong directional consistency with the initial IVW findings. In order to strengthen the validity of this relationship, a reverse Mendelian randomization (MR) analysis was conducted, which yielded no evidence of reverse causality.

Prognostic analysis indicated that gastric cancer patients with elevated blood levels of arginine had a favorable prognosis. And, the multivariate COX analysis revealed that only arginine emerged as an independent prognostic factor, irrespective of variables such as sex, age, stage, differentiation, and other amino acids. This study also revealed that arginine exhibited superior prognostic predictive ability compared to traditional tumor markers such as CEA, CA199, CA72-4, as well as the nomogram model based on arginine demonstrated enhanced prognostic predictive ability. The AUC values for negative value of arginine at 1-year, 3-year, and 5-year intervals were 0.732, 0.771, and 0.972, respectively. The AUC values for CEA were 0.521, 0.570, and 0.424, for CA199 were 0.563, 0.600, and 0.588, and for CA72–4 were 0.580, 0.672, and 0.605 at 1-year, 3-year, and 5-year intervals. The AUC values for the nomogram model were 0.957, 0.960, and 0.961 for 1-year, 3-year, and 5-year predictions, respectively. In recent years, arginine has been identified as a sensitive marker for breast and prostate cancer (16, 17), as well as a prognostic marker for gastric cancer in our previous study (12). In this study, we further found its independent prognostic value in gastric cancer.

Research has demonstrated that arginine possesses the ability to modulate the anti-tumor functionality of T cells, thereby enhancing patient survival (18). Supplementation of arginine has been shown to ameliorate the tumor immune microenvironment and bolster anti-tumor immune responses (19). Furthermore, investigations have revealed a correlation between arginine levels in the bloodstream and the efficacy of immunotherapy in cancer patients, with those exhibiting higher arginine concentrations displaying heightened sensitivity to immunotherapy and prolonged survival rates (20).

In accordance with prior research conducted by Nanthakumaran et al. (14), arginine has been demonstrated to exert significant growth-inhibitory and apoptosis-inducing effects on gastric cancer cells at concentrations ranging from 2 to 32 mM. This concentration range is deemed clinically attainable as physiological or therapeutic serum levels *in vivo* following arginine supplementation (14). Our findings corroborate these results, showing that arginine effectively inhibits the proliferation of gastric cancer cells within this specified concentration range. In the present study, a sublethal concentration of 12.5 mM was selected for transcriptomic sequencing to capture robust gene expression changes under high-arginine exposure. However, it must be emphasized that 12.5 mM is a supra-physiological, pharmacological concentration. For subsequent transcriptomic sequencing, a sublethal concentration of arginine was selected, revealing that the differentially expressed genes (DEGs) were predominantly associated with cellular metabolism signaling pathways and protein binding functions. This observation suggests a potential mechanism by which arginine exerts its protective effects. Conversely, it is important to note that in normal physiological conditions, where arginine does not reach such elevated levels, its protective roles may be mediated through alternative physiological processes or homeostatic mechanisms, as previously described.

In conclusion, our analysis of genetically predicted plasma arginine levels in relation to gastric cancer risk, alongside the assessment of patient plasma arginine levels and gastric cancer prognosis, as well as the examination of the functional effects of elevated arginine concentrations on gastric cancer cells, collectively suggest that arginine exerts a protective effect on both the risk and prognosis of gastric cancer. Prospectively, arginine shows significant potential to be employed as a preventive agent and a dependable prognostic marker for gastric cancer.

## Limitation

Due to the retrospective nature of this cohort, data on certain confounding variables—such as *Helicobacter pylori* infection status, smoking history, alcohol consumption, baseline BMI, and family history of malignant tumors—were unavailable in our primary clinical database and could not be adjusted for in the multivariable Cox model. The concentration of L-arginine (12.5 mM) used in our *in vitro* assays is significantly higher than normal physiological plasma levels (80–120 µM). Although this high-dose treatment successfully activated key downstream pathways as a proof of concept, it may not fully reflect the dose-dependent responses present at clinically relevant concentrations (e.g., 0.1–2 mM). Future studies incorporating physiological concentration gradients as well as *in vivo* model validations are still needed.

## Data Availability

The original contributions presented in the study are included in the article/**Supplementary Material**. Further inquiries can be directed to the corresponding author.

## References

[1] Joshi SS Badgwell BD . Current treatment and recent progress in gastric cancer. CA Cancer J Clin. (2021) 71:264–79. doi: 10.3322/caac.21657 33592120 PMC9927927

[2] Cocetta V Ragazzi E Montopoli M . Links between cancer metabolism and cisplatin resistance. Int Rev Cell Mol Biol. (2020) 354:107–64. doi: 10.1016/bs.ircmb.2020.01.005 32475471

[3] Lukey MJ Katt WP Cerione RA . Targeting amino acid metabolism for cancer therapy. Drug Discov Today. (2017) 22:796–804. doi: 10.1016/j.drudis.2016.12.003 27988359 PMC5429979

[4] Safrhansova L Hlozkova K Starkova J . Targeting amino acid metabolism in cancer. Int Rev Cell Mol Biol. (2022) 373:37–79. doi: 10.1016/bs.ircmb.2022.08.001 36283767

[5] Jiang Y Tao Q Qiao X Yang Y Peng C Han M . Targeting amino acid metabolism to inhibit gastric cancer progression and promote anti-tumor immunity: a review. Front Immunol. (2025) 16:1508730. doi: 10.3389/fimmu.2025.1508730 40018041 PMC11864927

[6] Hope HC Salmond RJ . The role of non-essential amino acids in T cell function and anti-tumour immunity. Arch Immunol Ther Exp (Warsz). (2021) 69:29. doi: 10.1007/s00005-021-00633-6 34637000 PMC8510955

[7] Zhang Y Hu H Liu W Yan SM Li Y Tan L . Amino acids and RagD potentiate mTORC1 activation in CD8(+) T cells to confer antitumor immunity. J Immunother Cancer. (2021) 9. doi: 10.1136/jitc-2020-002137 33883257 PMC8061841

[8] Sekula P Del Greco MF Pattaro C Köttgen A . Mendelian randomization as an approach to assess causality using observational data. J Am Soc Nephrol. (2016) 27:3253–65. doi: 10.1681/asn.2016010098 27486138 PMC5084898

[9] Birney E . Mendelian randomization. Cold Spring Harb Perspect Med. (2022) 12. doi: 10.1101/cshperspect.a041302 34872952 PMC9121891

[10] Zhang Y Li R Zhang S Li H . Association between non-neoplastic bladder diseases and bladder cancer risk: insights from Mendelian randomization studies. Postgrad Med J. (2025) 101:156–62. doi: 10.1093/postmj/qgae121 39287940

[11] Davies NM Holmes MV Davey Smith G . Reading Mendelian randomisation studies: a guide, glossary, and checklist for clinicians. BMJ. (2018) 362:k601. doi: 10.1136/bmj.k601 30002074 PMC6041728

[12] Shi LY Wang YY Jing Y Xu MH Zhu ZT Wang QJ . Abnormal arginine metabolism is associated with prognosis in patients of gastric cancer. Transl Cancer Res. (2021) 10:2451–69. doi: 10.21037/tcr-21-794 35116560 PMC8797619

[13] Ong JS MacGregor S . Implementing MR-PRESSO and GCTA-GSMR for pleiotropy assessment in Mendelian randomization studies from a practitioner's perspective. Genet Epidemiol. (2019) 43:609–16. doi: 10.1002/gepi.22207 31045282 PMC6767464

[14] Nanthakumaran S Brown I Heys SD Schofield AC . Inhibition of gastric cancer cell growth by arginine: molecular mechanisms of action. Clin Nutr. (2009) 28:65–70. doi: 10.1016/j.clnu.2008.10.007 19036482

[15] Lieu EL Nguyen T Rhyne S Kim J . Amino acids in cancer. Exp Mol Med. (2020) 52:15–30. doi: 10.1038/s12276-020-0375-3 31980738 PMC7000687

[16] Hu L Gao Y Cao Y Zhang Y Xu M Wang Y . Association of plasma arginine with breast cancer molecular subtypes in women of Liaoning province. IUBMB Life. (2016) 68:980–4. doi: 10.1002/iub.1581 27797142

[17] Selvi I Basar H Baydilli N Murat K Kaymaz O . The importance of plasma arginine level and its downstream metabolites in diagnosing prostate cancer. Int Urol Nephrol. (2019) 51:1975–83. doi: 10.1007/s11255-019-02261-8 31444697

[18] Geiger R Rieckmann JC Wolf T Basso C Feng Y Fuhrer T . L-Arginine modulates T cell metabolism and enhances survival and anti-tumor activity. Cell. (2016) 167:829–842.e13. doi: 10.1016/j.cell.2016.09.031 27745970 PMC5075284

[19] Zhang J Wang S Guo X Lu Y Liu X Jiang M . Arginine supplementation targeting tumor-killing immune cells reconstructs the tumor microenvironment and enhances the antitumor immune response. ACS Nano. (2022) 16:12964–78. doi: 10.1021/acsnano.2c05408 35968927

[20] Peyraud F Guégan JP Bodet D Nafia I Fontan L Auzanneau C . Circulating L-arginine predicts the survival of cancer patients treated with immune checkpoint inhibitors. Ann Oncol. (2022) 33:1041–51. doi: 10.1016/j.annonc.2022.07.001 35850444

